# Designing Anti-Viral Vaccines that Harness Intrastructural Help from Prior BCG Vaccination

**DOI:** 10.33696/immunology.5.174

**Published:** 2023

**Authors:** Tony W. Ng, Steven A. Porcelli

**Affiliations:** 1 Department of Microbiology and Immunology, Albert Einstein College of Medicine, Bronx, NY10461, USA; 2 Department of Medicine, Albert Einstein College of Medicine, Bronx, NY 10461, USA

**Keywords:** Vaccine, Antibody, Virus, Th cells, BCG, Linked recognition, Intrastructural help

## Abstract

Vaccines are among the most effective tools for combatting the impact and spread of infectious diseases. However, the effectiveness of a vaccine can be diminished by vaccine inequality, particularly during severe outbreaks of infectious diseases in resource-poor areas. As seen in many developing countries that lack adequate healthcare infrastructure and economic resources, the acquisition and distribution of potentially life-saving vaccines may be limited, leading to prolonged suffering and increased deaths. To improve vaccine equity, vaccine design must take into consideration the logistics needed to implement a successful vaccination drive, particularly among the most vulnerable populations. In the manuscript titled “Exploiting Pre-Existing CD4^+^ T Cell Help from Bacille Calmette-Guérin Vaccination to Improve Antiviral Antibody Responses” published in the Journal of Immunology, the authors designed a recombinant subunit vaccine against the Ebola virus (EBOV) glycoprotein that can harness the pre-existing T helper cells from prior BCG vaccination. As a recombinant subunit vaccine adjuvanted with alum, this approach has many features that make it well suited for the design of vaccines for developing nations, such as relative ease of production, scalability, and distribution. In addition, the high prevalence of BCG immunization and natural immunity to mycobacteria in many regions of the world endow such vaccines with features that should increase potency and efficacy among populations residing in such regions. As a result of using the helper activity of pre-existing BCG-specific Th cells to drive antibody responses, a lower vaccine dose is needed, which is a major advantage for vaccine manufacture. Furthermore, the BCG-specific Th cells also stimulate immunoglobulin class switching to IgG isotypes that have strong affinities for activating Fc-gamma receptors (FcγRs). Taken together, we propose that the design of subunit vaccines with intrastructural help from BCG-specific Th cells can improve protection against viral infection and represents a vaccine design that can be generally adapted to other emerging viral pathogens for the control and prevention of infection in many developing countries.

## Commentary

As the human population continues to expand and its footprint dominates the planet, disease outbreaks will become more intense and frequent. Although vaccination is one of the most effective methods against the devastation inflicted by infectious diseases, its unequal distribution, especially in developing and low resource continues to present a major limitation. This was clearly evident in the recent Coronavirus Disease 2019 (COVID-19) pandemic, in which the Severe Acute Respiratory Syndrome Coronavirus-2 (SARS-CoV-2) killed an estimated 7 million individuals worldwide, particularly with a higher burden afflicted in low-income countries [1,2]. It was estimated that distribution of the COVID-19 mRNA vaccine throughout the population in developed nations was achieved within 1 year, whereas for poorer countries there was approximately 3 years of lag time [3,4], undoubtedly inflicting millions of more deaths than it was necessary. In terms of infectious disease control, this issue of vaccine equity is a major concern, especially for the less wealthy nations. To minimize vaccine inequality and to rapidly stop the spread of deadly diseases, increased effectiveness in vaccine distribution is essential. In this regard, subunit vaccines remain one of the simplest forms of vaccines that are easily mass-produced and have stability without the need for low-temperature storage, which are both important features for improving logistics in vaccine distribution. However, a major drawback for subunit vaccines is the requirement for a strong adjuvant in the vaccine formulation to increase the immunogenicity and efficacy of the vaccine. Enormous efforts have been invested in adjuvant development to discover and produce adjuvants that elicit strong and protective immune responses [5,6]. This has been a major challenge in vaccine design, particularly because many strong adjuvants also induce unwanted side effects and may not be suitable for use in mass vaccination of human populations [7]. There is currently just a handful of FDA-approved adjuvants available for vaccine formulation in humans [8], and many of these do not elicit protective immune responses when formulated with subunit vaccines and require adjustments in dose and formulation to be compatible with different antigens [9]. We have studied the possibility of using the activities of pre-existing mycobacteria-specific helper T cells (Th) to address this problem [10]. Our previous studies took advantage of BCG Th cells, which are already present in BCG-vaccinated individuals, and increased the immunogenicity of the subunit vaccine, in this case, an Ebola virus glycoprotein, by adding BCG CD4^+^ T cell (Th) epitopes to the immunogen. This recombinant subunit vaccine containing the BCG Th epitopes (Th vaccine) is able to rapidly expand and recruit the pre-existing BCG-specific memory Th cells to provide the intrastructural help needed to shape the outcome of the antibody response without the need for complex adjuvants. Here we summarize the design of this vaccination strategy with respect to BCG vaccination, the types of antibodies elicited by the vaccine, and methods to further improve this vaccine strategy.

## The BCG Vaccine

**Figure 1.**
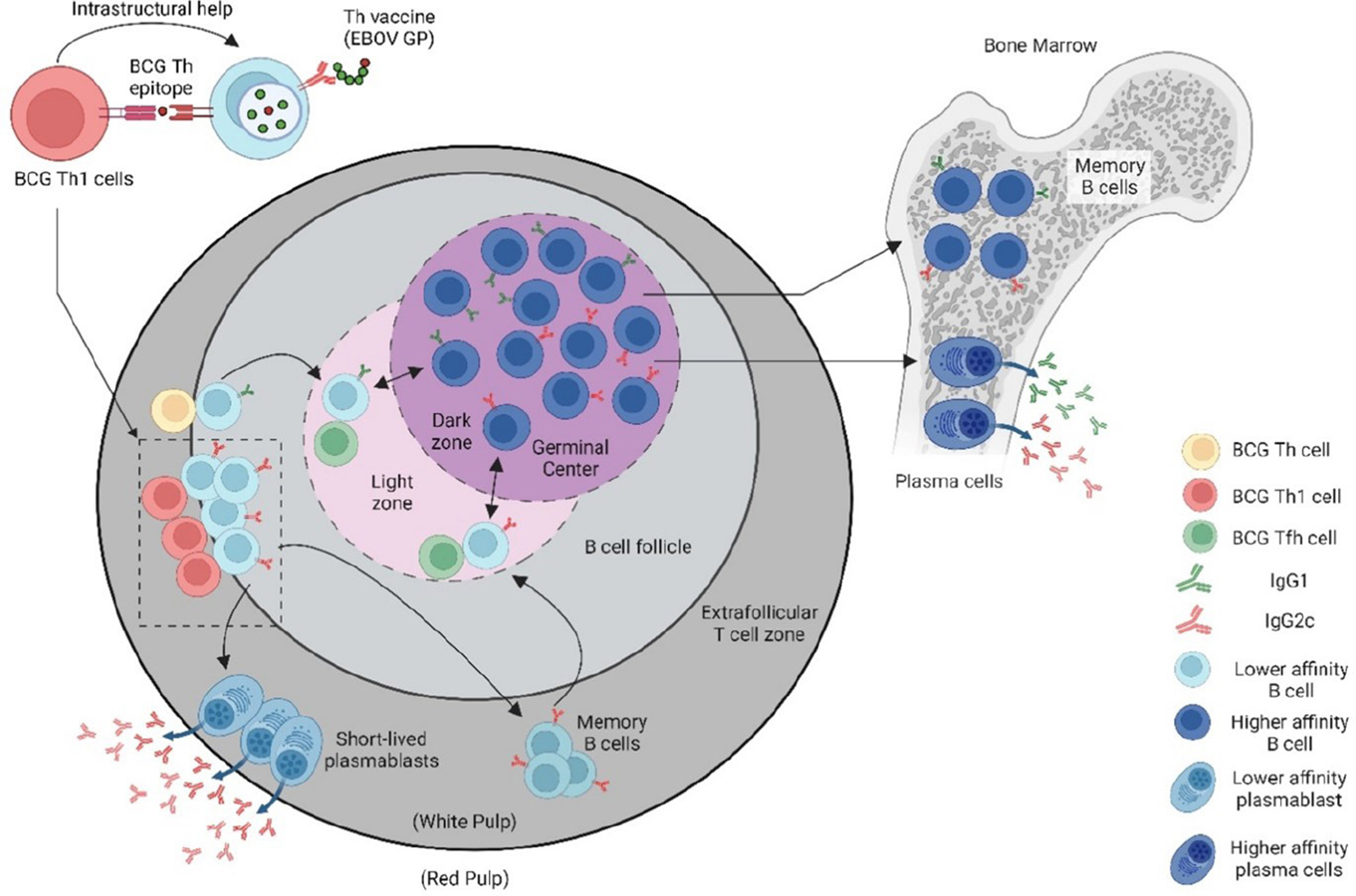
A vaccine designed with intrastructural help (recombinant subunit Th vaccine) to recruit pre-existing BCG Th cells for the development of protective antibodies against emergent viral pathogens. To take advantage of the pre-existing mycobacteria-specific T helper (Th) cells in BCG-vaccinated individuals, who often reside in regions of the world with high prevalence for emerging viral pathogens, a recombinant viral vaccine was designed in which the viral immunogen is fused to the mycobacteria Th cell epitope (P25) of Ag85B. Presentation of the P25 epitope by B cells specific for the viral antigen allows for cognate interactions with P25 Th cells to rapidly and potently promote virus-specific antibody responses. (Figure made in Biorender.com).

Bacille Calmette-Guérin (BCG), the only approved vaccine against *Mycobacterium tuberculosis* (Mtb), the etiological agent of human tuberculosis (TB), was developed in the late 19^th^ century by Drs. Albert Calmette and Camille Guérin [11] and has been administered to people throughout the world since 1924. Although the efficacy of the BCG against adult pulmonary TB remains controversial [12], it has been generally accepted that the vaccine does offer protection against childhood meningitis and miliary forms of TB [13]. On this basis, BCG is administered to approximately 100 million people every year as part of the universal vaccination policy for countries with high risk to TB infections [14]. With this extensive BCG vaccine coverage (>95% in many developing countries), many individuals have pre-existing BCG-specific memory helper T cells (Th). By providing BCG Th epitopes that correspond to immunodominant BCG antigens, the Th vaccine can rapidly recall the BCG specific memory Th cells in BCG vaccinated individuals to provide intrastructural help and potentially supplement weak viral Th responses that could limit antibody responses (Figure 1). As a result, the recruitment of BCG Th cells allows the induction of antibody responses with a significantly lower dose of the Th vaccine, which translate to reduced manufacturing demands and cost for mass production of the vaccine. BCG vaccination is a potent inducer of an inflammatory response that results in predominantly a Th1 subset [15] where IFNγ is known to activate antibody IgG class-switching [16]. These BCG Th1 cells induce antibodies with a range of effector functions, particularly those with IgG isotypes that favor binding to activating Fc receptors (FcγRs). Thus, the subunit Th vaccine design is able to take advantage of pre-existing BCG specific Th1 cells to lower vaccine dose and produce IgG class-switched antibodies that potentially carry out antibody-mediated effector functions.

Besides being used as a TB vaccine, BCG is also used to treat other diseases and infections not related to tuberculosis. A clinically important example is the use of the BCG vaccine as immunotherapy to treat non-muscle invasive bladder cancer [17,18]. A broader protection against infectious disease other than TB has also been attributed to BCG vaccination, including malaria [19], neonatal sepsis [20], and viral infections [21,22]. The specific mechanism of these off-target effects by BCG against other infections is not clear but it is often thought to involve trained immunity, which is the reprogramming of hematopoietic stem cells of the innate immune system through epigenetic changes. This constitutes a less specific form of immunological memory in which, after returning to basal levels following the initial stimulation by BCG, a subsequent stimulation by a different pathogen will induce a stronger response characterized by increased expression of proinflammatory cytokines, activation markers, and toll-like receptors [23–25]. However, many recent studies failed to show that trained immunity from BCG vaccination protects against COVID-19 [26–28]. Thus, while there are hints in many studies supporting the role of trained immunity in protection against various non-tuberculous diseases, the evidence is not strong enough to motivate changes in any current vaccination strategies [29,30]. The inconsistency of the protection conferred by trained immunity against different diseases may reflect the short duration of the memory of the innate immune response, the lack of specificity, and the mismatch between the innate and adaptive immunity activated by different pathogens. For example, if a major part of the trained immunity effect of BCG is the increased expression of activating FcγR on NK cells, this may only be effective against certain viral infections that induce the corresponding antibody isotype with high affinity to the Fc receptor. Since the increase in FcγR expression on innate immune cells is in part due to the effects of IFNγ [31], the IFNγ provided by the expansion of BCG Th1 cells by the Th vaccine may be involved in increasing the FcγR expression on innate cells while promoting IgG class-switching. These effects could synergize to promote antibody-mediated effector functions, including antibody-dependent cellular cytotoxicity (ADCC) and antibody-dependent cellular phagocytosis (ADCP), which are mechanisms that protect against certain viral infections.

## Vaccines against Viral Pathogens

The induction of neutralizing antibodies is generally considered the gold standard in vaccine development against viral pathogens. However, the generation of such antibodies often requires more extensive vaccine design and compositions, including the use of more complex adjuvants [32]. Furthermore, neutralizing antibodies focus mainly on the receptor binding sites (RBS) of viral glycoproteins as targets [33], therefore limit the number of epitopes available on the immunogen. Antibodies that mediate effector functions through their Fc domain can bind to any surface exposed targets, including epitopes outside of the RBS, which significantly increases the availability of epitopes on the immunogen to initiate cytolytic killing of infected cells by innate immune cells such as macrophages, neutrophils, and NK cells. It is well established that CD4^+^ T cells promote CD8^+^ T cell immunity [34]. Cytolytic CD8^+^ T cells specific for the viral epitopes can also be enhanced as part of the antiviral response by the Th vaccine through the help provided by BCG-specific CD4^+^ Th responses recruited by the BCG-specific Th epitopes in the Th vaccine. Thus, the Th vaccine approach not only promotes antibody-mediated effector killing of infected cells through IgG class-switching and upregulation of FcγR, but also enhances the adaptive immunity of anti-viral CD8^+^ T cells responses, making this a powerful multifaceted vaccine that can enhance the cytolytic functions of both the innate and adaptive immune systems.

## Improving BCG Th1 Responses

Many countries throughout the world lack a robust health care infrastructure to effectively control the spread of TB, especially through the use of expensive and prolonged TB drug treatments [35]. The only alternative method is implementing universal BCG vaccination to control, to some extent, TB infections [36]. Although the BCG-specific Th1 cells induced by the BCG vaccine are important for the control of Mtb infection, they alone are not sufficient to fully suppress disease or clear the infection [37]. Numerous efforts have been made to develop a TB vaccine or a vaccination strategy that can outperform the century-old suboptimal BCG vaccine. Many, if not all, of these TB vaccination strategies, such as BCG revaccination [38], administration of BCG through the intravenous route [39], or using attenuated Mtb mutants [40–44] resulted in increased immunogenicity, particularly with respect to the Th1 response toward mycobacterial antigens, which will enhance the potency of the BCG-dependent Th vaccine. The pipeline of TB vaccine research for the next few decades will most likely remain focused on enhancing the cellular immunity against Mtb [45,46], strategically putting the Th vaccine in a position to maximize the impact of capturing pre-existing T cell help for vaccinating against viral pathogens.

BCG has been used for many years as immunotherapy for treatment of non-muscle-invasive bladder cancer with a success rate of approximately 40% [47]. To improve the efficacy of BCG against bladder cancer, other immunotherapy approaches are included in the treatment, including monoclonal antibody therapy [48]. Preclinical studies in mice have shown synergistic antitumor effects on murine ascitic hepatoma cells by using BCG to improve the antibody therapy through activation of ADCC [49]. However, clinical trials (NCT00006352, NCT00003023) targeting various cancers (neuroblastoma, sarcoma, and small cell lung cancer) showed no impact on the outcome of disease responding to combination of monoclonal antibody therapies and BCG treatment [50]. More recently, several clinical trials (NCT03504163, NCT03711032, and NCT02138734) have been initiated to evaluate treatment of bladder cancers using monoclonal antibody therapy (pembrolizumab) or immunotherapy (ALT-803, an IL-15 superagonist] [51] together with BCG treatment. The Th vaccine, although it was initially designed to target viral antigens, could be easily modified by replacing the immunogen with a tumor antigen. This could provide a potent method for stimulating anti-tumor antibody responses to improve cancer immunotherapies.

## Discussion

The broad activities of the Th vaccine design described here include the increase in antibody titers to inhibit entry of pathogens into the host cell, the induction of IgG isotypes that activate Fcγ receptors to recruit effector functions against viral infections, and the ability of BCG-specific CD4^+^ T cells to support enhanced anti-viral CD8^+^ T cell responses. The Th vaccine design can be easily adopted to target a range of pathogens and diseases, which may be especially relevant during outbreaks when rapid production and distribution of vaccines is of key importance. Thus, the most apparent applications of the BCG driven Th vaccine design that we describe here are currently in the area of targeting endemic or emerging viral pathogens. Some obvious targets of current interest for this approach include the glycoprotein of Ebolavirus, the protein E of Zika virus, and the hemagglutinin of Influenza viruses. However, other possibilities for this vaccine strategy can be envisioned, including the augmentation of antibody dependent immune responses against tumors or a wider range of microbial pathogens. The fusion of weakly immunogenic antigens with BCG epitopes may be an effective strategy for increasing the levels and duration of antibody responses against key antigens identified as the “Achilles heel” of some of the stealthiest infectious microbes. Examples of interest in this regard could be the RH5 antigen of *Plasmodium falciparum* [52] and OspA protein of *Borrelia burgdorferi* [53].

## Conclusion

The Th vaccine design provides the flexibility for constructing vaccines that could be especially applicable for vaccination of populations with high prevalence of pre-existing immunity to BCG or other mycobacteria, such as often found in many developing countries. The Th vaccine is uniquely applicable to scenarios requiring rapid induction of antibody responses, such as during disease outbreaks caused by highly transmissible emerging viral pathogens. By incorporating the immunodominant BCG Th epitopes for recruitment of BCG-specific Th1 cells, the immunogenicity of the Th vaccine is increased to significantly reduce the effective vaccine dose and provide adjuvant effects that favor IgG class-switching important for mediating effector functions such as ADCC. The simplicity and adaptability of the Th vaccine design has the potential to vaccine production more affordable, facilitate distribution, and significantly level the inequality of vaccine availability that currently remains a major barrier in global prevention of infectious diseases.
